# Eggshell Quality Traits and Transcriptome Gene Screening Between Yunnong and Jingfen Chicken Breeds

**DOI:** 10.3390/biology13121048

**Published:** 2024-12-14

**Authors:** Zijian Li, Hao Wu, Jing Fu, Maida Mushtaq, Muhammad Khan, Yong Liu, Zobia Azeem, Hongmei Shi, Yang He, Ru Zhang, Muhammad Aziz Ur Rahman, Jiajia Kang, Changrong Ge, Kun Wang

**Affiliations:** 1 College of Animal Science and Technology, Yunnan Agricultural University, Kunming 650201, China; lzj01071217@163.com (Z.L.); 17686511136@163.com (H.W.); 18826485012@163.com (Y.H.); kjj730248@163.com (J.K.); gcrzal@126.com (C.G.); 2 Yunnan Animal Science and Veterinary Institute, Panlong District, Kunming 650201, China; fj11260027@163.com (J.F.); maida.ch17@gmail.com (M.M.); ruzhangru@163.com (R.Z.); 3 Yunnan Rural Revitalization Education Institute, Yunnan Open University, Kunming 650101, China; lydzq05091025@163.com (Y.L.); shm1219@126.com (H.S.); 4 Department of Zoology, University of Veterinary and Animal Sciences, Lahore 54000, Pakistan; zobiabajwa01@gmail.com; 5 Institute of Dairy Science, University of Agriculture, Faisalabad 03802, Pakistan; drazizurrahman@uaf.edu.pk

**Keywords:** Yunnon chicken, Jingfen chicken, eggshell, gene sequencing, enriched pathways

## Abstract

The eggshell quality traits in avian species, including Yunnong chickens (YN), are crucial for commercial and breeding purposes. The use of advanced biotechnologies, such as gene sequencing, has become increasingly common for identifying genetic markers and comparing specific traits in livestock. However, genetic markers related to eggshell traits in YN chickens remain unexplored. This study aimed to compare eggshell quality traits and identify genetic markers in YN chickens versus commercial Jingfen chickens (JF). A total of 400 chicks (200 per breed) were reared for 300 days under controlled conditions. At the end, 60 eggs from each breed were analyzed using scanning electron microscopy to examine eggshell cross-sections and mammillary layer structure. Tissue samples from kidneys and eggshell glands were collected from six hens per breed for RT-qPCR analysis to study gene expression profiles. Results showed that YN chickens had significantly higher eggshell strength, thickness, mammillary density, and effective layer thickness than JF chickens (*p* < 0.01). Despite this, YN chickens had a thinner mammillary layer. RT-qPCR analysis confirmed five candidate genes related to eggshells (LSS, NSDHL, MSMO1, SQLE, and FDFT1) that play an important role in this process. Pathway analysis revealed significant enrichment in several biological processes, including steroid biosynthesis, glycerolipid metabolism, purine metabolism, and thiamine metabolism. Based on these results, the YN chickens have strong eggshells with better thickness, mammillary density, and effective layer thickness. In addition, genomic and pathways analysis suggest a complex interaction of metabolic, immune, and cellular processes potentially influencing eggshell quality traits in poultry.

## Simple Summary

The Yunnong (YN) chicken is a local Chinese breed known for its egg-laying capabilities. This study aimed to identify genes associated with eggshell quality in YN chickens by comparing them with the commercially established Jingfen (JF) breed. The results showed that YN chicken eggs had superior eggshell thickness, strength, mammillary density, and effective layer thickness compared to JF eggs. RT-qPCR analysis identified five key genes—LSS, NSDHL, MSMO1, SQLE, and FDFT1—linked to eggshell quality. Pathway enrichment analysis revealed a complex interplay of metabolic, immune, and cellular processes potentially influencing eggshell traits in poultry.

## 1. Introduction

The Yunnong chicken (YN) breed, primarily found in Yunnan Province, China, is valued for its high-quality egg production. They are distinctive with their single black comb, black beak, and mostly black skin with some white patches, black tibiae, and colorful feathers in shades of black, white, and yellow. These chickens begin laying eggs at 123–140 days and produce 220–250 eggs annually, with an average weight of 40–45 g and an eggshell thickness of 0.38 to 0.45 mm. On the other hand, Jingfen chickens (JFs) are a commercial chicken breed known for high egg production capacity (over 280 eggs/annum) and widely utilized in the China poultry industry due to their efficiency in converting feed into eggs and consistent performance under intensive farming conditions. Despite China’s significant egg production in 2022 (18.84 million tons) [1], eggshell strength remains a challenge, causing economic losses during collection and transportation.

Eggshell breakage accounts for 8–11% of total losses [2]. Breeding programs aimed at improving eggshell quality have proven highly complex mechanisms involved in eggshell transformation [3]. The eggshell is a bioceramic with properties closely tied to its reproductive functions [4]. From a reproduction perspective, eggshells provide a stable environment for embryonic development and supply calcium ions essential for embryo growth [5]. In addition, eggshell quality influences the degree of damage during transport and processing, reducing financial losses. It also serves as a natural barrier against microbial contamination, lowering food safety risks [6]. A study by Roberts and Chousalkar [7] revealed that caged and free-range eggs had overall lower internal microbial contamination, suggesting the effectiveness of eggshells as a barrier. So far, it can be concluded that poor eggshell negatively impacts hatchability and increases embryonic mortality, further harming the industry’s economic viability [8].

Advances in modern nutrition and environmental controls have addressed issues of nutrient imbalance, but differences in eggshell formation under high-intensity production conditions remain key to influencing shell quality [9]. Studies exploring alternative protein sources, such as legumes, have shown that egg weight, yolk weight, and eggshell weight, strength, and thickness tend to increase with the hen’s age [10,11]. While traditional quality indicators include eggshell strength, thickness, and egg shape, little research has focused on material attributes like microstructure, which plays a critical role in shell quality. In 2004, the International Chicken Genome Sequencing Consortium published the genome sequence of *Gallus gallus*, the first non-mammalian organism to have its genome sequenced [12]. The chicken genome comprises approximately 1 billion base pairs and 20,000–23,000 genes, providing insights into vertebrate genome evolution and enhancing mammalian genome annotation [12]. Zhang and Deng [13] found that vector-based transient receptor vanilloid-6 (TRPV6) shRNA can inhibit the expression of vanilloid-6 in chicken osteoblasts. TRPV6 regulates the expression of calbindin-D28K during calcium transport. Additionally, Zhu and Li [14] found that dietary cadmium supplementation (30 and 60 mg/kg) significantly reduced eggshell cuticle thickness and roughened the shell surface while reducing the expression of calbindin1 (CALB1), ovocalyxin-32 (OCX-32), ovocalyxin-36 (OCX-36), osteopontin (SPP1), and ovocledidin-17 (OC-17). Ovocleidin-116 (OC-116), a homolog of the avian mineralo-phospho glycoprotein (MEPE), is part of a protein family known as small integrin-binding ligand N-linked glycoproteins (SIBLINGs) [15]. In chickens, OC-116 is found in the eggshell matrix and is expressed in uterine cells. It has also been linked to bone mineralization in mammals [16]. Significant research has focused on improving eggshell quality through dietary supplementation, mainly by adjusting trace elements like calcium and phosphorus, optimizing vitamin D levels, and incorporating other additives to strengthen shells [17,18]. These nutritional interventions aim to enhance calcium absorption and subsequently improve shell integrity. The importance of calcium, phosphorus, and vitamin D3 in eggshell quality is well documented [19]. However, recent studies investigated eggshell quality from a microstructural or genetic perspective [20,21]. Although transcriptomic sequencing is widely used in livestock production, there are few studies on the differential expression of genes affecting eggshell quality in laying hens [21]. This gap makes hens an ideal model for investigating the molecular mechanisms behind eggshell formation. Keeping all this in view, the current experiment aimed to examine the comparative eggshell quality between YN and JF chickens and the screening of eggshell quality-associated genes. In addition, our objective was to identify differentially expressed genes associated with signaling pathways that influence eggshell quality, highlighting genetic differences between the two breeds using RNA-sequencing techniques.

## 2. Materials and Methods

### 2.1. Experimental Animals and Feeding Management

The protocols and procedures of this experiment were approved by the animal use and care committee of Yunnan Agriculture University (YAU), Kunming, China, before the research trial (Protocol ID: YAUACUC01). In this experiment, a total of 400-day-old chicks, comprising 200 of the YN breed and 200 of the JF breed, were obtained from the teaching hatchery of Yunnan Agricultural University (Kunming, China) and reared in a controlled environment until 300 days of age. The selected chicks of these two breeds (n = 200 chicks per breed) were divided into four groups (n = 100 chicks/group and 2 groups/breed) following a completely randomized design. The groups were subjected to a phase feeding program, which included a basal diet from 1 to 100 days of age (rearing phase) and a laying diet from 43 to 300 days of age (laying phase). The feed formulations and their chemical compositions (on a dry matter basis) are given in Table 1. The feed formulation was in accordance with the established standards of the research poultry farm of Yunnan Agriculture University.

The chicks were raised in floor pens with litter during the brooding period (42 days). Before the experiment, the brooding area was thoroughly cleaned, scrubbed with a 10% copper sulfate solution, whitewashed, and disinfected using a 10% formalin solution. Drinkers, feeders, and feeding trays used during brooding were cleaned, washed, dipped in a 4% potassium permanganate (KMnO4) solution, and sun-dried. Sawdust was procured from a local vendor, sieved using a 4 mm particle separator, and evenly layered at a depth of 5.08 cm across the pens. Chick papers, feeders, and drinkers were placed in each pen, and the airtight shed was fumigated for 24 h using a combination of KMnO4 (17.5 g) and formalin (35 mL). The shed temperature was gradually reduced from 32 °C to 20 °C, in accordance with the birds’ comfort, while maintaining an average relative humidity of 50.3% during the brooding period. The lighting program consisted of 24 h of light at 30 lux for the first 7 days, followed by 18 h of light at 10 lux until day 42.

After the rearing phase, the hens were transferred to an individual H-Type-4-Tier 2-Door Layer Battery Cage System (dimensions 1200 mm × 600 mm × 430 mm) and reared until 300 days of age. Feed was offered twice daily (at 6:00 and 18:00), with 5% orts adjusted daily to ensure ad libitum intake. Fresh, clean water was available at all times. During the egg production phase, the light intensity was maintained at 0.5-foot candles, with 16 h of light provided daily. The birds were immunized against viral infections according to the vaccination schedule detailed in Table 2. After 56 days of age, the vaccination schedule was followed based on the established standards of the research poultry farm of Yunnan Agriculture University.

### 2.2. Sample Collection

At 300 days of age, 30 eggs from each group (n = 60 for the YN breed and n = 60 for the JF breed) were selected by considering their body weights (homogeneous) for egg quality analysis. Additionally, three hens from each group (n = 6 hens in total for each breed) were randomly chosen, fasted for 16 h, and humanely slaughtered via cervical dislocation, following the Chinese national standards for laboratory animals. Eggshell gland and kidney tissues were collected into sterile, RNase-free centrifuge tubes, immediately frozen in liquid nitrogen, and stored at −80 °C for further analysis.

### 2.3. Egg Quality Analysis

The collected eggs were evaluated within one day after collection for egg quality parameters, including longitudinal diameter (mm), transverse diameter (mm), egg weight (g), egg shape index (ratio between transverse and longitudinal diameters), eggshell thickness (mm), eggshell strength, egg white height (mm), yolk color, Haugh unit, yolk weight (g), and eggshell weight (g). Eggshell strength was measured using an eggshell strength tester (Robotmation Co., Ltd., Tokyo, Japan). Egg weight (g), white height (g), Haugh unit, and yolk color were assessed using a fully automatic multi-function egg quality analyzer (TOUHOKU RHYTHM Co., Ltd., Saitama, Japan).

### 2.4. Microstructure Analysis

After the egg quality assessment, the eggshells of 12 eggs (n = 6 eggs/breed) were retained and air-dried at room temperature for 24 h. Two fragments of the eggshell (0.5–1 cm^2^) from the equatorial region were collected, washed with double-distilled water to remove surface contaminants, and air-dried at room temperature. The samples were then immersed overnight in a solution containing 6% sodium hypochlorite, 4.12% sodium chloride, and 0.15% sodium hydroxide, followed by washing with distilled water and air drying at room temperature [22]. One fragment was mounted vertically on the sample stage of the scanning electron microscope (KYKY Technology Co., Ltd.; Beijing, China) for observation of the eggshell’s cross-sectional structure, and a total 3 scans/sample was taken at 500× to measure the total eggshell thickness, effective layer thickness (combined palisade, vertical crystal, and cuticle sections), and mammillary layer thickness. The mammillary thickness was taken as the length from the top of the membrane to the bottom of the palisade layer. The other fragment was mounted flat on the sample stage and scanned 3 times/sample at 500× for the examination of the eggshell’s mammillary structure and to determine mammillary density. The total eggshell thickness, effective layer thickness, and mammillary density (numbers of mammillary knobs per unit) were then measured and calculated by using ImageJ software (1.45 version; Wisconsin, DE, USA). The thickness of the mastoid layer was measured from the basal caps of the mammillary knobs to the point at which the palisade columns first fused.

### 2.5. Total RNA Extraction, Purification, RNA-Seq, and Annotation

The collected eggshell gland (JF-ZG and YN-ZG) and kidney (JF-S and YN-S) tissue samples were thawed, and RNA was extracted using the TIANGEN kit (TIANGEN, Beijing, China), following the manufacturer’s instructions. RNA purity was assessed using a Nanodrop 2000 spectrophotometer (Nanodrop, Wilmington, DE, USA), with OD260/OD280 values of between 1.8 and 2.1, indicating high RNA purity and the absence of significant impurities, making the samples suitable for further experiments. The total RNA concentration was measured using a Qubit 2.0 Fluorometer (Thermo Fisher Scientific, Waltham, USA), and RNA integrity was evaluated with an Agilent 2100 Bioanalyzer (Agilent Technologies, Santa Clara, CA, USA). PolyA-containing mRNA from the total RNA was enriched using Oligo (dT) magnetic beads. The RNA was then fragmented to approximately 300 bp using ionic fragmentation. The first strand of cDNA was synthesized using the fragmented RNA as a template with a 6-base random primer and reverse transcriptase. The second strand of cDNA was synthesized using the first strand as a template. Next-generation sequencing (NGS) was performed on these libraries using the Illumina HiSeq sequencing platform, employing paired-end (PE) sequencing.

### 2.6. Validation of RNA-Seq Results via RT-qPCR

Quantitative PCR was performed on RNA extracted from 16 eggshell gland and kidney tissue samples using the SYBR Premix Ex Taq™ II RT-qPCR kit (Takara, Tokyo, Japan). The amplification reaction was prepared in a total volume of 20.0 μL, consisting of 10 μL of SYBR Premix Ex Taq II (2×), 1.0 μL of forward primer, 1.0 μL of reverse primer, 2.0 μL of template RNA, and 6 μL of double-distilled water. The amplification protocol involved 40 cycles, starting with a pre-denaturation step at 95 °C for 180 s, followed by denaturation at 95 °C for 30 s, annealing at the appropriate temperature for 30 s, and extension at 72 °C for 20 s. Data were analyzed for relative quantification using the 2^(−ΔΔCT)^ method, with GAPDH as the reference gene. The primers used for RT-qPCR are listed in Table 3.

### 2.7. Bioinformatics and Statistical Analysis

Raw data were cleaned and trimmed using FastQC (version 0.11.5, FastQC GitHub) and Trim Galore (version 0.4.4, Trim Galore GitHub), respectively. Clean data alignment was performed with HISAT2 (version 2.0.4, HISAT2 GitHub), and the aligned data were compared to the Gallus gallus genome (Gallus gallus genome) to obtain SAM files. These SAM files were converted to BAM files using Samtools (version 1.5, Samtools GitHub). Differential gene expression analysis was conducted using DESeq2 (DESeq2 GitHub). The adjusted *p*-value (padj, FDR) was used to indicate the significance of differentially expressed genes, while log2FoldChange (log2FC) represented the relative gene expression levels. Differentially expressed genes (DEGs) were defined based on log2FC ≥ 1 and FDR < 0.05 (15). The pathways associated with DEGs were analyzed using the Kyoto Encyclopedia of Genes and Genomes (KEGG) database, and potential biological functions were identified. Relative expression levels of candidate target genes were calculated from Cq values using qBase+ software (Version 3.0) (17). Egg quality and eggshell microstructure data were analyzed using Statview software (SAS Institute Inc, Cary, North Carolina, USA, Version 5.0.1.0). Means were separated using the Tukey test, with significance adjusted at *p* < 0.05.

## 3. Results

### 3.1. Egg and Eggshell Quality

The comparative values of quality-based parameter analysis between YN and JF chickens are presented in Table 4. The horizontal and vertical diameters of the JF group eggs were greater (*p* < 0.01) than those of the YN group. However, the shape index did not show significant differences (*p* > 0.05) across the groups. The eggshell strength and thickness were greater (*p* <0.01) for the YN group than for the JF group. However, the egg and eggshell weights of Yunnong chickens were lower (*p* < 0.01) than those of the Jingfen chickens. On the other hand, the YN group had higher (*p* < 0.01) values for yolk weight and color than the JF group. The values of egg white height and Haugh units were numerically higher for the YN group than for the JF group; however, the mean differences were not statistically significant (*p* > 0.05).

### 3.2. Correlation Between Egg Quality and Eggshell Parameters

The correlation analysis between egg quality and eggshell parameters is given in Table 5. According to Table 5, there is a significant (*p* < 0.01) correlation between different eggshell and egg quality parameters. Eggshell-associated parameters such as transverse diameter, egg shape index, eggshell strength, and eggshell thickness had a significant positive association with egg quality parameters, including yolk color, egg white height, and Haugh unit, as well as the correlations between eggshell strength and thickness, egg white height, and Haugh unit (*p* < 0.01).

### 3.3. Microstructure of Eggshell

**Figure 1:**
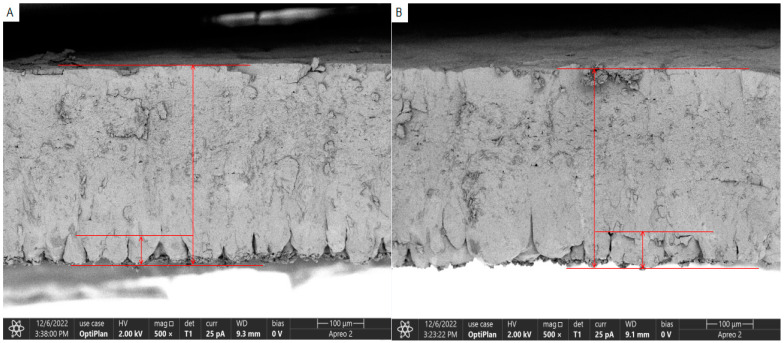
Cross-section microstructure of the YN chicken eggshell (**A**) and the JF chicken eggshell (**B**) at 500×. The larger area arrow represents the effective layer thickness, while the smaller one is the mastoid layer thickness.

**Figure 2:**
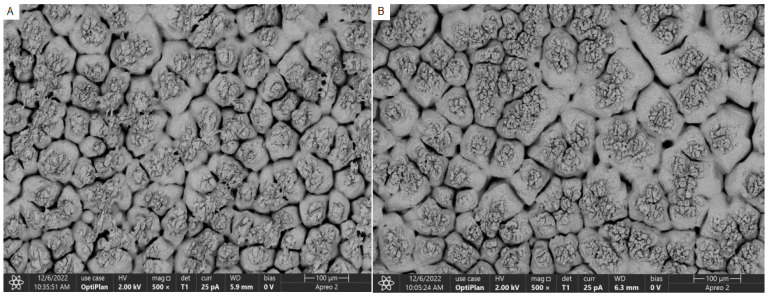
Microstructure of eggshell mammillary layer of the mastoid process of the YN chicken eggshell (**A**) and the JF chicken eggshell (**B**) at 500×.

The results of scanning electron microscopy of the eggshell cross-section and internal papillae of the eggs of both the YN and JF groups are presented in Figure 1 and Figure 2. The observed eggshell thickness was greater for the YN group than for the JF group, and the density of the mammillary layer in the shells of the YN group was higher than in those of the JF group. The measurements for eggshell thickness, effective layer thickness, mammillary layer thickness, and mammillary density are provided in Table 6. Specifically, the eggshell thickness, papillary density, and effective layer thickness of YN chickens were greater than those of the JF chickens, while the thickness of the mammillary layer in the YN group was smaller than that of the JF group (*p* < 0.01).

### 3.4. Transcriptome Analysis

**Figure 3:**
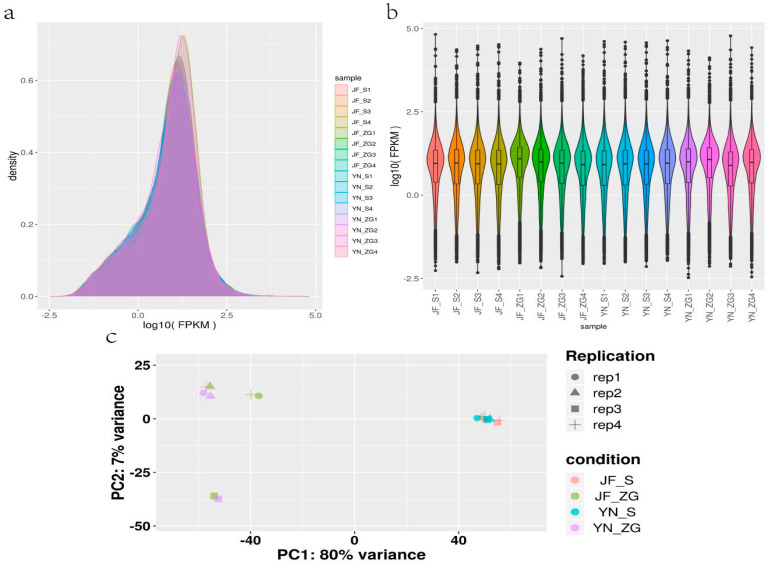
FPKM data expression density plot (**a**), density plot (**b**), and PCA cluster analysis plot (**c**) of kidney (JF_S, and YN_S) and eggshell gland (JF_ZG and YN_ZG) tissues of Yunnong and Jingfen chickens.

The kidney and eggshell gland tissue samples for the YN and the JF chickens were sequenced with four replicates in each group. The number of filtered raw reads ranged from 43,104,252 to 53,016,208 bp. The Q20 and Q30 proportions were greater than 97% and 93%, respectively. The total number of clean reads after quality control (QC) was between 42,253,146 and 53,016,208 bp (Tables S1 and S2). The base distribution results (Figure S1) indicate a preference in nucleotide composition for the first 10 bp, consistent with expected phenomena associated with the Illumina platform. The nucleotide content of A, T, G, and C at other positions remained stable and balanced, indicating a high overall quality suitable for subsequent bioinformatics analyses. We utilized Bowtie2 to construct a reference genome index and aligned the filtered reads to the reference genome using Tophat2. The statistics of the sequence alignment are summarized in the table provided. The percentage of total sequences aligning with the reference genome ranged from 91.36% to 93.21%, with sequences aligning with multiple positions constituting 2.02% to 3.54%. Furthermore, the percentage of sequences aligning uniquely with a single position was between 96.46% and 97.98%, demonstrating a high alignment rate of the reads on the genome (Table S3). Comparing the gene expression levels of YN and JF kidney and eggshell gland tissues, more than 70% of the genes showed expression levels that were more than double. FPKM data from all samples were used to create expression density plots (Figure 3a) and FPKM plots (Figure 3b), with results showing that expression levels were generally consistent. The FPKM values of the 16 samples were analyzed using a PCA diagram (Figure 3c), which revealed that samples from the same tissues of the same variety clustered together, indicating similar genetic backgrounds.

### 3.5. RNA-Seq Differential Expression Profiling

**Figure 4:**
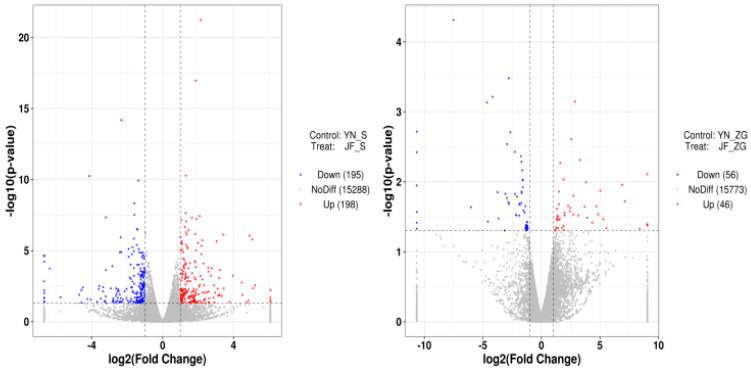
Volcano plot of differentially expressed genes of Yunnong and Jingfen chicken breeds in kidney (JF–S and YN–S) and eggshell gland (JF–ZG and YN–ZG) tissues.

**Figure 5:**
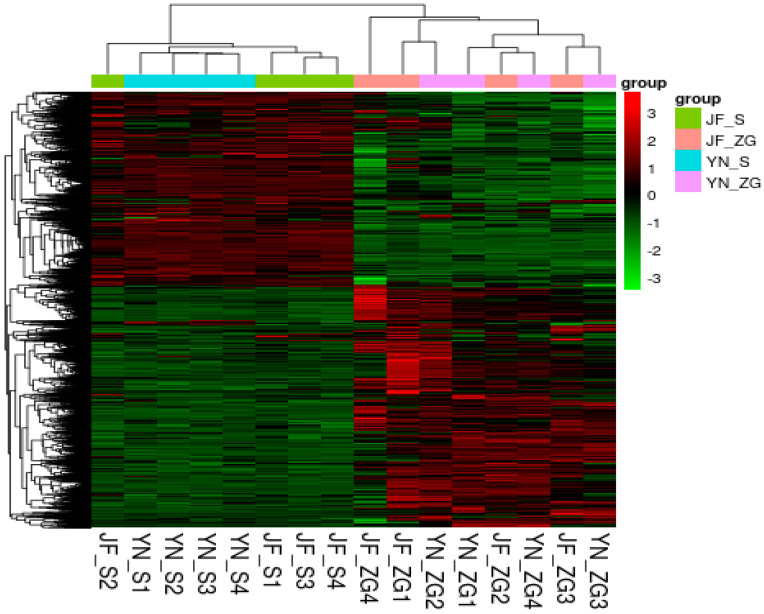
Cluster map of differentially expressed genes between Yunnong and Jingfen chicken breeds in kidney (JF–S and YN–S) and eggshell gland (JF–ZG and YN–ZG) tissues.

The differential expression analysis between the YN–ZG and JF–ZG groups and between the YN–S and JF–S groups was performed using DESeq. The criteria for identifying differentially expressed genes were set to [log2FoldChange] > 1 and *p* < 0.05, resulting in a total of 495 differentially expressed genes. As shown in Table 7, the comparison between YN–ZG and JF–ZG identified 102 differential genes, of which 56 were upregulated and 46 were downregulated. In contrast, the comparison between YN–S and JF–S revealed 393 differentially expressed genes, with 195 upregulated and 198 downregulated. Volcano plots illustrating the differentially expressed genes for both comparisons are presented in Figure 4, alongside a clustering heatmap of the differential expression genes shown in Figure 5.

### 3.6. Gene Enrichment Analysis

**Figure 6:**
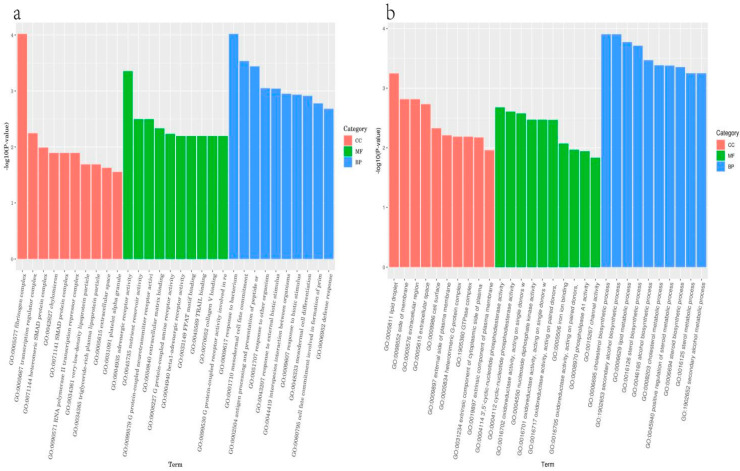
GO enrichment analysis between the YN–ZG_JF–ZG group (**a**) and the YN–S_JF–S group (**b**).

To investigate the transcriptional changes and biological processes of genes in laying hens during egg production, we conducted a Gene Ontology (GO) enrichment analysis using the topGO package (Figure 6). This analysis involved calculating gene lists and counts for each GO term based on the annotated differentially expressed genes, followed by determining *p*-values using the hypergeometric distribution method, with significant enrichment defined as *p* < 0.05. This approach identified GO terms that were significantly enriched in the differentially expressed genes compared to the entire genomic background. The results of the GO enrichment analysis were categorized into three domains: biological process (BP), cellular component (CC), and molecular function (MF). In the comparison between the YN–ZG and JF–ZG groups, a total of 2237 entries were enriched, with 364 entries significantly enriched. In contrast, the comparison between the YN–S and JF–S groups revealed a total of 4587 enriched entries, of which 548 were significantly enriched (Figure 6). Additionally, we performed KEGG enrichment analysis to identify significantly enriched pathways associated with the differentially expressed genes, with a focus on pathways related to egg quality and eggshell formation. As presented in Table 8, the comparison between the YN–ZG and JF–ZG groups highlighted significantly enriched pathways, including steroid biosynthesis, phototransduction, TGF-beta signaling pathway, ether lipid metabolism, and vascular smooth muscle contraction. The comparison between the YN–S and JF–S groups revealed similar significantly enriched pathways, which included steroid biosynthesis, glycerolipid metabolism, purine metabolism, influenza A, thiamine metabolism, biosynthesis of unsaturated fatty acids, PPAR signaling pathway, cytosolic DNA-sensing pathway, neuroactive ligand–receptor interaction, and the p53 signaling pathway. These findings underscore the relevance of specific signaling pathways to both egg quality and eggshell characteristics.

### 3.7. Validation of DEGs by RT-qPCR

**Figure 7:**
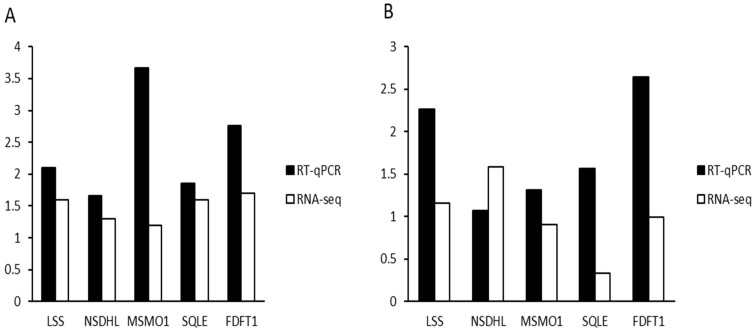
Comparison between RT-qPCR and RNA-seq results for the eggshell glands (**A**) and kidney (**B**) tissues of the Yunnong and Jingfen chicken breeds.

In this study, RT-PCR was employed to verify the expression of differentially expressed genes between the two groups. For the comparison groups YN–ZG vs. JF–ZG and YN–S vs. JF–S, the relative expression levels of five genes LSS, NSDHL, MSMO1, SQLE, and FDFT1 were analyzed using RT-qPCR and compared with the RNA-seq results. As illustrated in Figure 7, the RT-qPCR results and RNA-seq data exhibited identical expression trends for each gene in both comparison groups, confirming the accuracy of the RNA sequencing data.

## 4. Discussion

Eggshell quality assessment is significant for a variety of purposes, including chicken farming, food safety, and genetic research [23]. Eggshell quality, along with other essential production factors such as egg size or lay rate, is critical in the chicken breeding selection phase [24]. Better eggshell quality indicates that chickens have efficient calcium metabolism, which translates to good utilization of dietary calcium [25]. So far, eggshell quality assessment and the identification of associated genetic markers have enabled breeding programs focused on boosting shell strength, thickness, and overall durability. It is commonly understood that eggshell gives calcium and other nutrients to the developing embryo [26]. As a result, poor eggshell quality can impede embryo development and reduce hatchability rates in breeding operations. Furthermore, stronger and more homogeneous eggshells are less likely to break or become contaminated, extending the shelf life of eggs and increasing consumer safety [27]. The eggshell also acts as the first line of defense against bacterial contamination, notably diseases like Salmonella [27]. The eggshell structure-associated parameters can directly influence mechanical properties and quality assessment parameters. Different methods including scanning using electron microscopy to examine the eggshells being used across the world for both commercial as well as research purposes [23]. In this study, the eggshell thickness and strength of Yunnong chickens were found to be greater than those of Jingfen chickens. Ultrastructural cross-section observations revealed that the mammillary layer of Yunnong chickens was thinner compared to that of Jingfen chickens. Additionally, internal ultrastructural analysis indicated that the mammillary density in Yunnong chickens was higher than that in Jingfen chickens. In summary, Yunnong chickens demonstrate superior eggshell thickness and strength compared to Jingfen chickens. Earlier research established that the mammillary layer was the most common site of structural alteration in cracked eggs [28]. Additionally, a study by Liao and Qiao [4] reported a significant positive correlation between the percentage thickness of the mammillary layer and eggshell strength. However, Bain [29] suggested that the role of the mammillary layer in shell strength is bidirectional. Furthermore, pigment deposition is another factor influencing eggshell strength [30]. Pigment granules stored in the shell gland epithelial cells reach their maximum deposition rate at the end of eggshell calcification [31], which may impair the deposition of the vertical crystal layer, leading to reduced density and a more porous structure. As mineral deposition in the eggshell decreases, the eggshell matrix proteins may be upregulated, potentially affecting eggshell quality and reducing eggshell strength. Moreover, trace elements such as zinc, manganese, and copper serve as cofactors for certain enzymes and can influence the mechanical properties of eggshells by affecting the formation of calcite crystals and altering the crystalline structure of the eggshell [32]. Early experiments indicated that hens fed a manganese-deficient diet produced thinner eggshells, with alterations in the microstructure of the mammillary layer and reduced levels of hexosamines and hexuronic acids in the organic matrix [33]. This study also found that Yunnong chickens exhibited highly significant positive correlations between various metrics, including the egg shape index and eggshell strength, eggshell strength and eggshell thickness, eggshell strength and the Haugh unit, and egg white height and the Haugh unit. These findings align with the results of Bogdanski and Silveira [34] and Wang and Ruan [35].

Several genes have been implicated in the biomechanical properties and formation of eggshells. For example, single nucleotide polymorphisms (SNPs) in the ovocleidin-116 (OC-116) gene are associated with the modulus and thickness of eggshells [36]. The ovocleidin-17 (OC-17) gene is predicted to catalyze the conversion of amorphous calcium carbonate into calcite, the primary component of mineralized eggshells [37]. Additionally, SNPs in the OCX-32 gene have been shown to significantly correlate with the thickness of the mammillary layer in birds, while osteopontin (OPN) is associated with eggshell fracture toughness [38]. In this study, candidate differential genes related to eggshells were identified through transcriptome sequencing analysis, revealing significantly enriched KEGG pathways, including steroid biosynthesis, light signal transduction, TGF-beta signaling, ether lipid metabolism, and vascular smooth muscle contraction. The five most representative genes identified were LSS (lanosterol synthase), NSDHL (NAD(P)-dependent steroid dehydrogenase-like), MSMO1 (methylsterol monooxygenase 1), SALE (squalene epoxidase), and FDFT1 (farnesyl diphosphate farnesyltransferase 1). However, research on the roles of LSS, NSDHL, and FDFT1 in animals is relatively scarce. Liu and Liu [39] identified key genes related to steroid metabolism, such as LSS, by analyzing the pectoral muscle of chickens from high-triglyceride-content (HTG) and low-triglyceride-content (LTG) groups. In humans, mutations in the LSS gene can lead to congenital cataracts and alopecia [40], while its effects in animals are typically associated with fat deposition [41]. Zhang and Li [42] utilized quantitative real-time PCR to confirm that NSDHL knockdown increased the proliferation of 3T3-L1 preadipocytes while diminishing their differentiation, suggesting that NSDHL is a novel regulator of lipogenesis. Iannaccone and Elgendy [43] reported that the expression of genes such as NSDHL was lower in Friesian calves fed diets supplemented with grape pomace (GPO) compared to those not receiving GPO, correlating with reduced cholesterol levels in the blood and showcasing antioxidant activity. While NSDHL is implicated in cancer development in humans, research on this gene in livestock and poultry remains limited. Guo and Wei [44] employed RNA-seq technology to identify differentially expressed genes in the subcutaneous adipose tissue of Muscovy ducks at three developmental stages (12 weeks, 35 weeks, and 66 weeks). Compared to the 12-week stage, the subcutaneous adipose tissue at 35 weeks exhibited upregulation of genes related to cholesterol and fatty acid biosynthesis, such as HSD17B7 and MSMO1, providing potential candidate genes and pathways involved in fat deposition in ducks and enhancing understanding of the molecular mechanisms governing fat deposition during development. Guo and Wei [44] investigated the effects of ovariectomy on meat quality in broilers; they found that ovariectomized birds showed significant increases in abdominal fat weight (AFW) and abdominal fat percentage (AFP) at 14 and 19 weeks post-surgery compared to the sham operation group. Genes such as CETP, DGAT2, DHCR24, HSD17B7, and MSMO1 were significantly upregulated following ovariectomy. In humans, mutations in the MSMO1 gene have been linked to diseases such as pancreatic cancer [45], whereas in livestock, MSMO1 appears to influence fat deposition in chickens [39] and ducks [46]. Zhang and Deng [47] transfected an SQLE overexpression vector into bovine skeletal muscle-derived mesenchymal stem/stromal cells (MSCs) and found that SQLE significantly promoted MSC differentiation and apoptosis while inhibiting proliferation, revealing its role in myoblast differentiation and providing new insights into the regulatory network of muscle development in cattle. To investigate the biological functions and regulatory roles of gga-miR-221-5p in chicken liver, Zhang and Wang [48] discovered that the highly conserved gga-miR-221-5p directly targets ELOVL6 and SQLE mRNAs, influencing intracellular triglyceride and total cholesterol levels. Furthermore, 7β-estradiol was shown to suppress the expression of gga-miR-221-5p while upregulating ELOVL6 and SQLE expression, thereby promoting triglyceride synthesis and cholesterol levels in the ovaries of egg-laying hens. In humans, SQLE is associated with the inhibition of colorectal cancer, and in livestock, it influences muscle development in cattle, with its expression in the chicken liver potentially impacting cholesterol deposition in the ovaries during the laying period. Huang and Zhang [49] identified FDFT1 as a potential biomarker associated with ferroptosis in kidney cancer (KIRC), finding that FDFT1 upregulation inhibited cell proliferation, migration, and invasion, with potential antitumor effects occurring via the AKT signaling pathway. Nie and Shan [50] analyzed the role of ferroptosis in the prognosis of colorectal cancer (CC) through RNA-seq, establishing a prognostic model composed of five ferroptosis-related genes (AKR1C1, ALOX12, FDFT1, ATP5MC3, CARS1), concluding that ferroptosis-related features could partially predict the survival rates of CC patients. In this study, LSS, NSDHL, MSMO1, SQLE, and FDFT1 were identified as significant genes derived from the steroid biosynthesis signaling pathway. Through transcriptome analysis, this study found that all five genes within the steroid biosynthesis signaling pathway were significantly upregulated and that this pathway was related to the synthesis of vitamin D3. Furthermore, the results were validated by quantitative real-time PCR, corroborating the findings from transcriptome sequencing. The expression levels of LSS, NSDHL, MSMO1, SQLE, and FDFT1 may be positively correlated with eggshell formation.

## 5. Conclusions

Based on the results, the comparison with Jingfen (JF) chickens demonstrates the superior eggshell strength and thickness in YN chickens, alongside a denser and more robust mammillary layer structure. The consistency between the RT-qPCR and RNA-Seq results strengthens the identification of the five candidate genes that could play a key role in regulating these traits. The LSS, NSDHL, MSMO1, SALE, and FDFT1 genes may regulate the expression of related genes by modulating calcium metabolism, thereby promoting calcium activation in the eggshell gland during eggshell formation. This process could lead to improvements in the eggshell microstructure and enhanced eggshell strength. By investigating the genes influencing eggshell strength and examining the underlying mechanisms at the microscopic level, this study offers new insights and a foundation for further research into the molecular pathways involved in eggshell formation.

## Figures and Tables

**Table 1:** Feed formulation and chemical composition on a dry basis.

Components	Period I	Period II
Corn (%)	63.25	67.22
Soybean meal (%)	30.25	18.84
Wheat bran (%)	0.00	10.00
Fishmeal (%)	2.51	0.00
Coarse stone powder (%)	0.39	0.46
Fine stone powder (%)	0.70	0.62
Dicalcium phosphate (%)	1.51	1.52
Methionine (%)	0.08	0.07
Salt (%)	0.35	0.31
^1^ Commercial premix (%)	1.00	1.00
Chemical composition on a dry basis		
Dry matter (%)	88.90	89.10
Crude protein (%)	20.20	18.02
ME (Kcal/kg DM)	3116.24	3020.12
Crude fiber (%)	3.67	4.02
Ether extract (%)	2.12	2.02
Ash (%)	3.44	3.02
Calcium (%)	0.74	0.97
Phosphorous (%)	0.34	0.41

^1^ Commercial premix contained Fe, 39 g; Mn, 36 g; Cu, 7.2 g; Zn, 25 g; I, 1.15 g; Cr, 0.12 g; Se, 0.18 g; Vit A, 23 MIU; Vit D3, 4.6 MIU; Vit E, 68.0 g; Vit K, 7.0 g; Vit B1, 6.0 g; Vit B2, 21.0 g; Vit B6, 6.0 g; Vit B12, 0.02 g; niacin, 64.0 g; calcium D pantothenate, 29.0 g; folic acid, 4.5 g; biotin, 0.24 g; and Vit C, 96.0 g.

**Table 2:** Vaccination schedule.

Vaccinations (Type)	Vaccination Age (Days)	Route
Malek’s vaccine	1	Subcutaneous (neck)
Combined Newcastle disease and infectious bronchitis vaccine	3	Eyes
Infectious bursal disease vaccine	12	Water
Combined Newcastle disease and infectious bronchitis vaccine + reassortant avian influenza virus vaccine	20	Subcutaneous (neck)
Infectious bursal disease vaccine	25	Water
Avian pox vaccine	35	Wings
Avian coryza trivalent vaccine	42	Muscle
Reassortant avian influenza virus	49	Muscle
Mycoplasma synovia vaccine	56	Muscle

**Table 3:** Primer used for RT-qPCR analysis.

Gene	Primer Sequence	Annealing Temperature (°C)
GAPDH	Forward: TAGTGAAGGCTGCTGCTGAT	60
	Reverse: AAGGTGGAGGAATGGCTGTC	
LSS	Forward: AGCATTGGTCCGATTTCC	60
	Reverse: TCGTACCCTGCATCTTCAT	
NSDHL	Forward: ATGATGAACCAATCCCTT	60
	Reverse: TTAGCAGCCACAGCACTA	
MSMO1	Forward: GAGGATGCCTGGCACTAT	60
	Reverse: CTCCAAGGATGAGCGTTT	
SQLE	Forward: AAGTTATTGTTGTGGGTTCA	60
	Reverse: AATCCTGTCAGGCTCCTT	
FDFT1	Farward: AGAGGGGTGGTGAAGATTCG	60
	Reverse: TGGCACAGGACAGGTAGATG	

**Table 4:** Egg quality comparison of Yunnong and Jingfen chicken breeds.

Item	Yunnong Chicken	Jingfen Chicken
LD ^1^	54.00 ± 2.00 ^A^	55.50 ± 1.67 ^B^
TD ^2^	40.85 ± 1.00 ^A^	42.24 ± 1.11 ^B^
EI ^3^	1.322 ± 0.050	1.315 ± 0.047
ESS ^4^	4.45 ± 0.16 ^A^	4.08 ± 0.06 ^B^
EST ^5^	0.429 ± 0.041 ^A^	0.362 ± 0.020 ^B^
EW ^6^	48.07 ± 8.09 ^A^	54.63 ± 3.66 ^B^
ProH ^7^	5.98 ± 1.06	5.69 ± 1.68
YC ^8^	14.30 ± 0.47 ^A^	10.64 ± 1.20 ^B^
HU ^9^	80.53 ± 10.40	75.12 ± 13.46
ESW ^10^	6.95 ± 0.45 ^A^	8.23 ± 0.96 ^B^
YW ^11^	17.32 ± 1.89 ^A^	13.12 ± 1.80 ^B^

^1^ LD = longitudinal diameter (mm), ^2^ TD = transverse diameter (mm), ^3^ EI = egg shape index, ^4^ ESS = eggshell strength, ^5^ EST = eggshell thickness (mm), ^6^ EW = egg weight (g), ^7^ ProH = protein height (mm), ^8^ YC = yolk color, ^9^ HU = Haugh unit, ^10^ ESW = eggshell weight (g), and ^11^ YW = yolk weight (g). Note: Different uppercase letters between different species of shoulder labels in the same row show significant differences (*p* < 0.05).

**Table 5:** Pearson correlation between egg quality and shell quality.

	LD ^1^	TD ^2^	EI ^3^	ESS ^4^	EST ^5^	EW ^6^	ProH ^7^	YC ^8^	HU ^9^	ESW ^10^	YW ^11^
LD ^1^	1.000										
TD ^2^	−0.101	1.000									
EI ^3^	0.043	−0.052	1.000								
ESS ^4^	0.031	0.031	0.917 **	1.000							
EST ^5^	0.062	−0.008	0.928 **	0.864 **	1.000						
EW ^6^	0.307	−0.179	−0.141	−0.155	−0.144	1.000					
ProH ^7^	0.071	−0.027	0.977 **	0.951 **	0.946 **	−0.152	1.000				
YC ^8^	0.109	0.473 **	0.009	0.129	−0.012	0.143	0.066	1.000			
HU ^9^	0.080	−0.043	0.935 **	0.909 **	0.931 **	−0.147	0.972 **	0.081	1.000		
ESW ^10^	0.320	−0.017	−0.044	0.021	−0.119	−0.035	−0.033	0.061	−0.072	1.000	
YW ^11^	0.124	0.139	−0.055	0.071	−0.041	0.110	−0.044	−0.058	−0.093	−0.039	1.000

^1^ LD = longitudinal diameter (mm), ^2^ TD = transverse diameter (mm), ^3^ EI = egg shape index, ^4^ ESS = eggshell strength, ^5^ EST = eggshell thickness (mm), ^6^ EW = egg weight (g), ^7^ ProH = protein height (mm), ^8^ YC = yolk color, ^9^ HU = Haugh unit, ^10^ ESW = eggshell weight (g), and ^11^ YW = yolk weight (g). Note: At *p*-level 0.05 (double-tailed), the correlation is significant. ** At *p*-level 0.01 (double-tailed), the correlation is significant.

**Table 6:** Egg quality comparison of Yunnong and Jingfen chicken breeds.

Item	YN Chicken	JF Chicken
Eggshell thickness (μm)	416.37 ± 10.25 ^A^	350.87 ± 9.93 ^B^
Mastoid thickness (μm)	58.82 ± 2.33 ^A^	69.06 ± 1.66 ^B^
Effective layer thickness (μm)	357.55 ± 15.79 ^A^	281.81 ± 7.79 ^B^
Mammillary density	130.40 ± 7.27 ^A^	111.20 ± 7.69 ^B^

Note: Different uppercase letters between different species of shoulder labels in the same row show significant differences (*p* < 0.05).

**Table 7:** Differentially expressed genes between Yunnong and Jingfen chicken breeds in the kidney (JF–S, and YN–S) and eggshell gland (JF–ZG and YN–ZG) tissues.

Control	Treatment	Up-Regulated	Down-Regulated	Total
JF–ZG	YN–ZG	56	46	102
JF–S	YN–S	195	198	393

**Table 8:** Statistical analysis of KEGG enrichment of Yunnong and Jingfen chicken breeds in the kidney (JF–S_YN–S) and eggshell gland (JF–ZG_YN–ZG) tissues.

Group	Name	Pathway ID	Gens
	Steroid biosynthesis	gga00100	MSMO1, NSDHL, FDFT1, SQLE, LSS
	Phototransduction	gga04744	RHO
YN–ZG_JF–ZG	TGF-beta signaling pathway	gga04350	THBS1, SMAD1, BAMBI
	Ether lipid metabolism	gga00565	GAL3ST1
	Vascular smooth muscle contraction	gga04270	ADRA1B, CALML3
	Steroid biosynthesis	gga00100	NSDHL, DHCR24, LSS
	Glycerolipid metabolism	gga00561	DGKI, LIPC, LIPG, LPIN1
	Purine metabolism	gga00230	PDE1B, PDE10A, GUCY2C, PDE6H, AK1
	Influenza A	gga05164	IL18, RSAD2, BLB1, CCL5, TNFRSF10B, MX1
YN–S_JF–S	Thiamine metabolism	gga00730	AK1
	Biosynthesis of unsaturated fatty acids	gga01040	FADS2, SCD
	PPAR signaling pathway	gga03320	ANGPTL4, FADS2, SCD
	Cytosolic DNA-sensing pathway	gga04623	CCL5, CCL4, IL18
	Neuroactive ligand–receptor interaction	gga04080	GABRA4, GZMA, APLNR, CALCA, AGT, SSTR3, GRIN2A, CALCR, GRIA4, GH
	p53 signaling pathway	gga04115	CDK1, AIFM2, TNFRSF10B, STEAP3

## Data Availability

The data supporting the findings of this study are available on request from the corresponding authors.

## Supplementary Materials

The following supporting information can be downloaded at: https://www.mdpi.com/article/10.3390/biology13121048/s1, Figure S1: Base distribution, Table S1: Library basic information, Table S2: Filtered data, Table S3: Comparison result statistics.
