# Supplementary material for: Eggshell Quality Traits and Transcriptome Gene Screening Between Yunnong and Jingfen Chicken Breeds
Source: Biology (Basel). 2024 Dec 14;13(12):1048. doi: 10.3390/biology13121048 (PMC11673107; doi:10.3390/biology13121048)
Supplement: Supplementary file 1 [file biology-13-01048-s001.zip › biology-3329618-supplementary.pdf]

Table S1. Library basic information

Sample	Reads number	Bases/bp	Q30/bp	N/%	Q20/%	Q30/%
JF_S1	43104252	6508742052	6153783594	0.001482	98.05	94.54
JF_S2	43881384	6626088984	6267562448	0.001490	98.05	94.58
JF_S3	51344958	7753088658	7291858433	0.001488	97.84	94.05
JF_S4	52764308	7967410508	7471190704	0.001490	97.73	93.77
JF_ZG1	48906958	7384950658	6971756797	0.001495	98.00	94.40
JF_ZG2	50484920	7623222920	7196163572	0.001482	97.93	94.39
JF_ZG3	52806970	7973852470	7503419835	0.001486	97.76	94.10
JF_ZG4	51308300	7747553300	7313773465	0.001491	98.01	94.40
YN_S1	46974976	7093221376	6694813486	0.001475	98.00	94.38
YN_S2	44895790	6779264290	6381888291	0.001495	97.90	94.13
YN_S3	48149436	7270564836	6839157736	0.001472	97.88	94.06
YN_S4	42253146	6380225046	6045447726	0.001492	98.11	94.75
YN_ZG1	53016208	8005447408	7567078339	0.001514	97.98	94.52
YN_ZG2	51006852	7702034652	7268653595	0.001468	97.94	94.37
YN_ZG3	46224740	6979935740	6514624565	0.001454	97.46	93.33
YN_ZG4	50857628	7679501828	7250760096	0.001478	98.01	94.41

Note: Sample: name of sample; Reads No.: Total number of Reads; Bases (bp): the total number of bases;Q30(bp):Tota l number of bases whose base recognition accuracy is above 99.9%;N(%):percentage of fuzzy bases; Q20(%):the perce ntage of bases with base recognition accuracy above 99%; Q30(%):The percentage of bases whose base recognition acc uracy is above 99.9%.

Table S2. Filtered data

Sample	Clean Reads Number	Clean Data /bp	Clean Reads/%	Clean Data/%
JF_S1	40840460	6166909460	94.74	94.74
JF_S2	41518474	6269289574	94.61	94.61
JF_S3	48622908	7342059108	94.69	94.69
JF_S4	50005414	7550817514	94.77	94.77
JF_ZG1	46282324	6988630924	94.63	94.63
JF_ZG2	47783454	7215301554	94.64	94.64
JF_ZG3	50020092	7553033892	94.72	94.72
JF_ZG4	48525230	7327309730	94.57	94.57
YN_S1	44427368	6708532568	94.57	94.57
YN_S2	42492800	6416412800	94.64	94.64
YN_S3	45598822	6885422122	94.70	94.70
YN_S4	39981828	6037256028	94.62	94.62
YN_ZG1	50066976	7560113376	94.43	94.43
YN_ZG2	48236286	7283679186	94.56	94.56
YN_ZG3	43788728	6612097928	94.73	94.73
YN_ZG4	48128650	7267426150	94.63	94.63

Note: Sample: Sample name; Clean Reads No: High-quality sequence reads; Clean Data(bp): the number of high-qual ity sequence bases; Clean Reads%: Percentage of high-quality sequence reads for sequencing reads; lean Data%: The percentage of high-quality sequence bases in sequencing bases.

Table S3. Comparison result statistics

Sample	Clean Reads	Total Mapped	Multiple Mapped	Uniquely Mapped
JF_S1	40840460	37919712(92.85%)	850452(2.24%)	37069260(97.76%)
JF_S2	41518474	38418410(92.53%)	870740(2.27%)	37547670(97.73%)
JF_S3	48622908	44758622(92.05%)	904192(2.02%)	43854430(97.98%)
JF_S4	50005414	46204089(92.40%)	985893(2.13%)	45218196(97.87%)
JF_ZG1	46282324	42557246(91.95%)	1197584(2.81%)	41359680(97.19%)
JF_ZG2	47783454	44200579(92.50%)	1136343(2.57%)	43064236(97.43%)
JF_ZG3	50020092	45697046(91.36%)	1231168(2.69%)	44465878(97.31%)
JF_ZG4	48525230	45426028(93.61%)	1010880(2.23%)	44415148(97.77%)
YN_S1	44427368	41165166(92.66%)	885928(2.15%)	40279638(97.85%)
YN_S2	42492800	39423314(92.78%)	796717(2.02%)	38626597(97.98%)
YN_S3	45598822	42332617(92.84%)	864015(2.04%)	41468602(97.96%)
YN_S4	39981828	37007703(92.56%)	770810(2.08%)	36236893(97.92%)
YN_ZG1	50066976	46439728(92.76%)	1162335(2.50%)	45277393(97.50%)
YN_ZG2	48236286	43986509(91.19%)	1141399(2.59%)	42845110(97.41%)
YN_ZG3	43788728	39656492(90.56%)	1404607(3.54%)	38251885(96.46%)
YN_ZG4	48128650	44861002(93.21%)	1107962(2.47%)	43753040(97.53%)

Note: Sample; Clean Reads: total number of sequences used for alignment; Total Mapped: Total sequence Mapped/Cl ean Reads for the reference genome; Multiple Mapped: Total number of sequences Mapped to Multiple locations. Per centage is multiple Mapped/total mapped; Uniquely Mapped: Total number of sequences aligned to one position onl y, percentages are Uniquely Mapped/total Mapped.

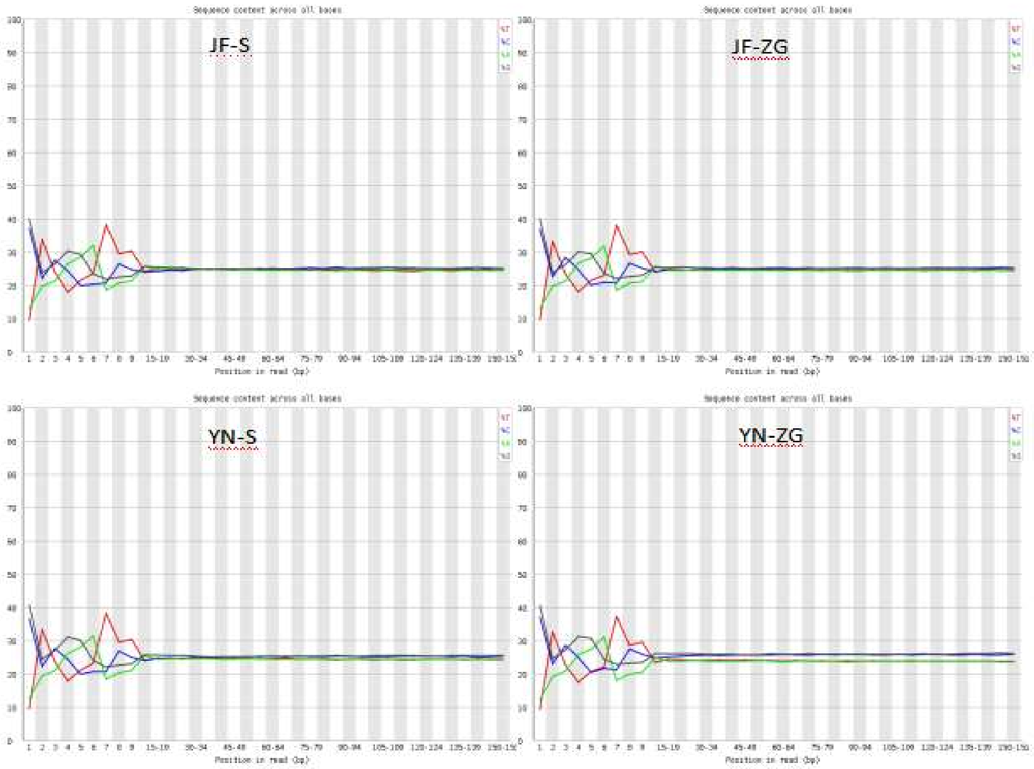

Figure S1: Base distribution.
